# CCN2/CTGF-Driven Myocardial Fibrosis and NT-proBNP Synergy as Predictors of Mortality in Maintenance Hemodialysis

**DOI:** 10.3390/ijms262311350

**Published:** 2025-11-24

**Authors:** Wen-Chin Ko, Che-Shao Chen, Yi-Ping Chang, Chi-Sheng Wu, Hung-Chi Yang, Jia-Feng Chang

**Affiliations:** 1 School of Medicine, Fu Jen Catholic University, New Taipei City 242062, Taiwan; 086938@mail.fju.edu.tw; 2 Division of Cardiac Electrophysiology, Department of Cardiovascular Center, Cathay General Hospital, Taipei 106438, Taiwan; 3 Department of Public Health, Institute of Epidemiology and Preventive Medicine, National Taiwan University, Taipei 106319, Taiwan; d12849010@ntu.edu.tw; 4 Department of Healthcare Information and Management, Ming Chuan University, Taoyuan Campus, Taoyuan 333321, Taiwan; d228@tyvh.gov.tw; 5 Division of Nephrology, Department of Internal Medicine, Taipei Veterans General Hospital, Taoyuan Branch, Taoyuan 330023, Taiwan; 6 Renal Care Research and Health Promotion Association, New Taipei City 220050, Taiwan; luke.chisheng@gmail.com; 7 Department of Medical Laboratory Science and Biotechnology, Yuanpei University of Medical Technology, Hsinchu 300102, Taiwan; hcyang@mail.ypu.edu.tw; 8 Department of Nursing, Yuanpei University of Medical Technology, Hsinchu 300102, Taiwan

**Keywords:** CCN2/CTGF, NT-ProBNP, cardiac remodeling biomarker, hemodialysis, composite risk score, mortality prediction model

## Abstract

Chronic inter-dialytic volume overload and uremic inflammation activate TGF-β/Smad3 and p38 MAPK pathways, inducing connective tissue growth factors (CCN2/CTGF)-mediated fibrosis and NT-proBNP secretion from over-stretched cardiomyocytes. The combined rise in CTGF and NT-proBNP reflects myocardial fibrosis, stiffness and remodeling, predicting cardiovascular (CV) death in maintenance hemodialysis (MHD) patients. From molecular pathology to clinical translation, circulating CCN2/CTGF and NT-proBNP levels and bio-clinical data among MHD patients were measured in this prospective cohort. Multivariate Cox regression analysis identified independent predictors of mortality, which were incorporated into a composite risk-score model. The predictive performance for all-cause, CV, and sudden cardiac death (SCD) was assessed using receiver operating characteristic (ROC) survival analysis. CCN2/CTGF, NT-proBNP, age, serum albumin, MHD vintage, high-sensitivity C-reactive protein, smoking, and diabetes mellitus were significant predictors. The integrated model yielded areas under the curve of 0.91 for all-cause mortality, 0.88 for CV mortality, and 0.87 for SCD. Integrated complementary biomarkers and clinical parameters significantly improve mortality risk prediction in MHD patients. This synergistic model provides clinicians with a robust tool for early CV screening, individualized intervention, and precision management for high-risk populations.

## 1. Introduction

Patients undergoing maintenance hemodialysis (MHD) experience recurrent interdialytic weight gain (IDWG) and chronic volume overload, driving progressive cardiovascular (CV) remodeling [1,2,3]. Thus, CV disease (CVD) remains the leading cause of death in this population [4,5]. Given CV remodeling and cardiomyopathy are central to cardiorenal mortality, developing reliable cardiac biomarkers and predictive models for patients with advanced chronic kidney disease (CKD) is imperative [6,7].

Connective tissue growth factor (CTGF) and N-terminal prohormone of brain natriuretic peptide (NT-proBNP) are dual biomarkers central to myocardial fibrosis, volume overload induced heart failure and fatal CV events [8,9]. CTGF, also known as cellular communication network factor 2 (CCN-2), regulates cell proliferation, differentiation, and extracellular matrix (ECM) homeostasis, contributing to the triad of inflammation, fibrosis, and hypertrophy [10,11]. Inhibition of CTGF in mouse models of myocardial infarction reduces hypertrophy and fibrosis, improving survival as potential therapeutic targets [12]. Elevated CTGF levels correlates with organ fibrosis, cardiorenal outcomes and mortality, supporting its utility for risk stratification and targeted intervention, especially in MHD patients with lifelong IDWG [13,14,15]. By understanding contemporary molecular biomarkers, clinicians can develop effective risk stratification tools and prediction models to identify therapeutic targets in cardiorenal care [16,17]. Among them, NT-proBNP released by cardiomyocytes is the most widely used biomarker to diagnose and monitor heart failure and risks of future CV events [18]. Through investigating combination effects of convincing prognostic biomarkers, there is a burst of interest in identifying novel scoring models that aim to more reliably predict events in high-risk patients [19,20,21].

Mechanistically, CTGF expression is primarily regulated by transforming growth factor-β (TGF-β)/Smad and mitogen-activated protein kinase (MAPK) signaling pathways, which are activated under mechanical stress, oxidative injury, and uremic toxin accumulation in MHD patients. Activation of TGF-β/Smad3 enhances CTGF transcription through Smad-binding elements within its promoter region, leading to fibroblast proliferation, α-smooth muscle actin (α-SMA) expression, and collagen type I deposition [22,23]. Moreover, CTGF interacts with integrin-linked kinase (ILK) and focal adhesion kinase (FAK), further amplifying downstream ERK and p38 MAPK cascades that promote extracellular matrix (ECM) accumulation and ventricular stiffening [24,25].

In parallel, NT-proBNP secreted from cardiomyocytes is primarily triggered by mechanical stress, which activates stretch-sensitive ion channels and downstream p38 MAPK–NF-κB and cyclic GMP–protein kinase G (PKG) signaling cascades. These pathways enhance BNP gene transcription and promote cardiomyocyte hypertrophy under sustained hemodynamic stress [26,27]. Emerging evidence also indicates that Ca^2+^/calcineurin–NFAT signaling may participate in stretch-induced transcriptional activation and cross-talk with PKG-mediated pathways in pressure-overloaded myocardium [27,28]. Notably, above mechanotransduction networks integrate mechanical, oxidative, and inflammatory cues, forming a shared mechano-fibrotic pathway that links CTGF and NT-proBNP responses to chronic volume overload. This convergence underscores their potential as complementary biomarkers reflecting fibrosis, hypertrophy, and hemodynamic stress in MHD-related cardiac remodeling.

Considering this, single biomarkers may not capture the complexity of disease processes, as they often reflect only one biological pathway. To overcome these limitations, a precise prediction model for the timely management of clinical events requires integration of CTGF, NT-proBNP and relevant bio-clinical factors. The comprehensive integration of multiple biomarkers into a single predictive model may enhance discrimination and reclassification, offering a foundation of artificial intelligence development to guide treatment decisions and improve outcomes [2,29]. Through Cox regression and receiver operating characteristic (ROC) analysis in stages to develop an optimal model, we aim to evaluate the predictive validity of a multivariate scoring system as an early warning tool for all-cause and CV mortality in MHD patients.

## 2. Results

### 2.1. Primary Analyses: Evaluation of the Clinical Candidate Predictors

The research completed medical records and follow-up in 168 MHD patients with baseline measurement. The comparison of bio-clinical data was summarized in Table 1, with respect to CTGF concentrations. Based on circulating CTGF concentrations, patients were divided into low (≤30.2 ng/mL) and high (>30.2 ng/mL) CTGF groups. Major significant differences between the groups were observed in age, DM prevalence, CVD history, all-cause mortality, CV death, hs-CRP and NT-ProBNP levels. Patients with CTGF > 30.2 ng/mL were older (73.6 ± 8.6 years) compared to those with CTGF ≤ 30.2 ng/mL (64.2 ± 6.9 years). Higher CTGF levels were associated with increased DM prevalence (54.0% vs. 35.8%) and CVD history (56.3% vs. 34.6%). These patients also had a longer HD vintage (77.6 ± 42.4 months vs. 66.6 ± 55.7 months), lower albumin levels (3.8 ± 0.5 g/dL vs. 3.9 ± 0.4 g/dL), higher incidence of all-cause mortality (7.4% vs. 35.6%) and CV death (4.9% vs. 24.1%), suggesting a potential impact of elevated CTGF on patient outcomes.

### 2.2. Univariate Cox Regression Analyses of Mortality Predictors

The univariate Cox regression analysis (Table 2) showed that older age, DM, smoking, longer HD vintage, lower albumin, higher levels of CTGF, NT-ProBNP and hs-CRP were significantly associated with all-cause mortality (HR: 1.065, 95% CI: 1.026–1.105, *p* < 0.01; 2.653 (1.350–5.214), *p* < 0.01; 2.143 (1.057–4.347), *p* < 0.05; 1.007 (1.001–1.012), *p* < 0.05; 0.191 (0.090–0.408), *p* < 0.001; 1.014 (1.005–1.022), *p* < 0.001; 1.003 (1.002–1.004), *p* < 0.001; 3.888 (2.755–5.486), *p* < 0.001, respectively). Age, DM, smoking, hypoalbuminemia, higher levels of CTGF, NT-ProBNP, and hs-CRP also significantly correlated with CV mortality (HR: 1.071, 95% CI: 1.023–1.121, *p* < 0.01; 3.007 (1.296–6.973), *p* < 0.05; 3.302 (1.479–7.370), *p* < 0.05; 0.390 (0.153–0.997), *p* < 0.05; 1.015 (1.006–1.025), *p* < 0.01; 1.004 (1.003–1.005), *p* < 0.01; 3.617 (2.366–5.530), *p* < 0.001, respectively). There was no statistically significant association between HD vintage and CV mortality (HR: 1.004, 95% CI: 0.997–1.011, *p* = 0.23), whilst HD vintage was strongly linked to all-cause mortality (HR: 1.007, 95% CI: 1.001–1.012, *p* = 0.01). Collectively, older age, hypoalbuminemia, longer HD vintage, DM, smoking, higher levels of CTGF, NT-ProBNP and hs-CRP consistently increase the risk of death.

### 2.3. Multivariate Cox Regression and Survival Analysis

Table 3 presents the results of the multivariate Cox regression analysis across three different models, each incorporating CTGF, NT-proBNP, and an additional clinical predictor (albumin, DM, or HD vintage) to assess their predictive value for both all-cause and CV mortality. In the model 1, both CTGF and NT-proBNP were significant predictors of all-cause mortality (aHR: 1.012, 95% CI: 1.000–1.023, *p* < 0.05; 1.003, 95% CI: 1.002–1.004, *p* < 0.01, respectively) and CV death (aHR: 1.018, 95% CI: 1.004–1.032, *p* = 0.01; 1.004, 95% CI: 1.003–1.005, *p* < 0.01, respectively). Albumin levels were significantly associated with a decreased risk of all-cause mortality (aHR: 0.286, 95% CI: 0.133–0.616, *p* < 0.01) but did not predict CV mortality (*p* = 0.309). In model 2, the inclusion of DM did not reach statistical significance for either all-cause mortality (*p* = 0.113) or CV mortality (*p* = 0.149). CTGF and NT-proBNP remained robust predictors for all-cause death (*p* < 0.05 and 0.01, respectively) and CV mortality (both *p* < 0.01). Likewise, HD vintage was not a significant predictor of either all-cause mortality (*p* = 0.236) or CV mortality (*p* = 0.912) in Model 3. After adjustment, CTGF and NT-proBNP were still associated with all-cause death (*p* < 0.05 and 0.01, respectively) and CV mortality (both *p* < 0.01). These findings confirm that CTGF and NT-proBNP are independent predictors of both all-cause and CV mortality across different covariate adjustment models.

**Figure 1:**
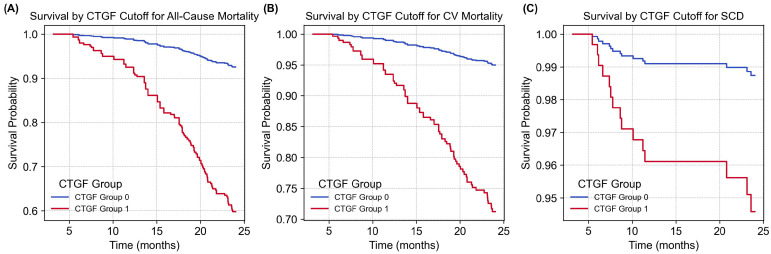
Cumulative survival curves of mortality risks with respect to cut-off concentrations of CTGF. Patients were divided into two groups based on cut-off concentrations of CTGF: group 0 (≤30.2 ng/mL) and group 1 (>30.2 ng/mL). (**A**,**B**) During 3682.0 person-months of follow-up, higher CTGF levels were linked to increased risks of all-cause mortality (*p* < 0.001) and CV mortality (*p* < 0.001). (**C**) The higher CTGF levels showed a statistically insignificant increase in risks for SCD (*p* = 0.187). The association between serum CTGF levels and SCD was not limited to higher CTGF levels, suggesting the necessity of developing a comprehensive risk scoring system. CTGF, connective tissue growth factor; CV, cardiovascular; SCD, sudden cardiac death.

Cumulative survival curves (Figure 1) display the impact of CTGF levels on mortality risks over 3682.0 person-months. Patients were classified into two groups based on CTGF levels: lower (≤30.2 ng/mL) and higher (>30.2 ng/mL). While higher CTGF levels were associated with an increased risk of all-cause mortality (aHR: 6.652, 95% CI: 2.770–15.974, *p* < 0.001) and CV mortality (aHR: 6.607, 95% CI: 2.262–19.296, *p* < 0.001), this model did not show a statistically significant increase in risk for sudden cardiac death (SCD) (aHR: 4.397, 95% CI: 0.487–39.722, *p* = 0.187). In light of this, CTGF alone is not strong enough to predict all CV events, suggesting the necessity of developing a comprehensive risk scoring system.

Supplementary Table S1 summarizes bio-clinical data and fatal events categorized by our multivariate risk scoring system, including significant risk factors of mortality in the univariate Cox regression analysis. Patients are stratified into five classes based on cumulative scores derived from their cut-off values in ROC analysis. According to the overall mortality prediction capability in the ROC analysis, the cut-off points (scores) for age, albumin, CTGF, hs-CRP, and HD months were 71 years (1 point), 3.7 g/dL (1 point), 30.2 ng/mL (1 point), 1.6 mg/L (1 point) and 58.5 months (1 point), respectively. The quartile for NT-ProBNP was as follows: ≥928.3 pg/mL (3 points); 825.5–496.2 pg/mL (2 points); 495.9–378.3 pg/mL (1 point); ≤377.8 pg/mL (0 point), respectively. After adding DM (1 point) and smoking (1 point), the total scores reach 10 points. Supplementary Table S1 presents a detailed comparison of bio-clinical parameters and fatal events stratified by the above integrated multivariate risk score classes. Significant differences in the distribution of various fatal events across different multivariate risk score classes highlight the prognostic value in assessing mortality risk among MHD patients.

**Figure 2:**
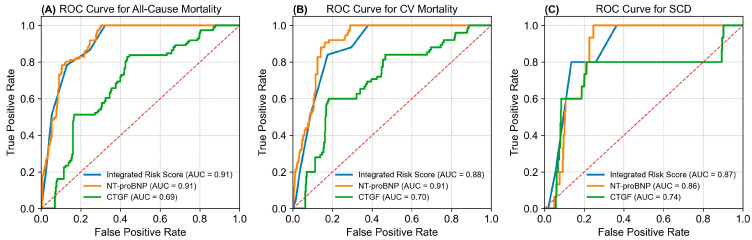
Comparisons of the integrated risk score, NT- ProBNP and CTGF levels for various mortality predictions using ROC analysis. The ROC analysis highlights the predictive power of the integrated risk score, NT-proBNP, and CTGF levels for various fatal events over 24 months. (**A**) All-cause mortality: AUCs were 0.91 for the risk score model, 0.91 for NT-proBNP, and 0.69 for CTGF. (**B**) CV mortality: AUCs were 0.88 for the risk score, 0.91 for NT-proBNP, and 0.70 for CTGF. (**C**) SCD: AUCs were 0.87 for the risk score, 0.86 for NT-proBNP, and 0.74 for CTGF. AUC = area under curve; CTGF = Connective Tissue Growth Factor; CV = cardiovascular; NT-ProBNP = N-terminal pro-brain natriuretic peptide. ROC = receiver operating characteristic. SCD = sudden cardiac death.

The secondary analyses focus on the evaluation of the three risk prediction models for all cause, CV death and SCD. Figure 2 draws a comparison of predictive values among CTGF, NT-ProBNP and the integrated multivariate risk score model using the ROC analysis over a 24-month follow-up period. NT-ProBNP demonstrates a strong predictive power, with an AUC of 0.912 (95% CI = 0.870 to 0.955) for all-cause mortality, 0.912 (95% CI = 0.868 to 0.957) for CV mortality, and 0.863 (95% CI = 0.739 to 0.936) for SCD. CTGF demonstrates a suboptimal predictive power, with an AUC of 0.694 (95% CI = 0.604 to 0.785) for all-cause mortality, 0.705 (95% CI = 0.597 to 0.812) for CV mortality, and 0.737 (95% CI = 0.456 to 1.000) for SCD. Our integrated multivariate risk score model also demonstrates a robust predictive power, with an AUC of 0.907 (95% CI = 0.854 to 0.948) for all-cause mortality, 0.883 (95% CI = 0.824 to 0.933) for CV mortality, and 0.877 (95% CI = 0.768 to 0.955) for SCD. Integrating CTGF and NT-ProBNP into our risk scoring system improves sensitivity and specificity for fatal outcome prediction. Compared with NT-ProBNP, our prediction model provides a better AUC in the ROC analysis for SCD. The integrated multivariate risk score system effectively identifies high-risk patients, enabling timely interventions in the MHD population.

### 2.4. Integrated Risk Score Model Summary

In the current study, we comprehensively evaluated bio-clinical predictors for all-cause and cardiovascular (CV) mortality using the Cox regression model as the primary analysis (Table 2). Older age, higher plasma concentrations of CTGF and NT-proBNP, longer hemodialysis (HD) vintage, diabetes mellitus (DM), smoking, higher hs-CRP, and lower albumin were all significantly associated with increased mortality risk. Based on these findings, we developed a novel death risk score system incorporating these pivotal predictors. The multivariate Cox regression analysis (Table 3) confirmed that CTGF and NT-proBNP were independent predictors of both all-cause and CV mortality. Figure 1 demonstrates the cumulative survival curves, showing that higher CTGF levels (>30.2 ng/mL) were associated with a significantly increased risk of all-cause and CV mortality (both *p* < 0.01).

In the secondary analyses, a comprehensive multivariate death risk prediction model was developed by integrating CTGF, NT-proBNP, and significant bio-clinical factors including age, HD vintage, DM, smoking, hs-CRP, and albumin. The integrated model showed superior predictive performance compared with single biomarkers. As illustrated in Figure 2, the composite risk score achieved an area under the curve (AUC) of 0.907 for all-cause mortality, 0.884 for CV mortality, and 0.877 for sudden cardiac death (SCD), outperforming CTGF or NT-proBNP alone. These results indicate that combining fibrosis-related (CTGF) and hemodynamic stress-related (NT-proBNP) biomarkers with clinical factors significantly enhances the predictive capacity for fatal outcomes in maintenance hemodialysis (MHD) patients.

Our current study identified CTGF and NT-proBNP as independent predictors of all-cause and CV mortality in MHD patients based on multivariate Cox regression. Older age, DM, smoking, hypoalbuminemia, higher CTGF, higher NT-proBNP and elevated hs-CRP were consistently associated with higher death risk. CTGF > 30.2 ng/mL was associated with significantly lower overall survival, demonstrating higher cumulative incidence of all-cause and CV death compared with CTGF ≤ 30.2 ng/mL groups. Although categorical CTGF did not significantly predict SCD based on Kaplan–Meier analysis (Figure 1), continuous CTGF remained a significant predictor of SCD in Cox modeling (Table 3). Given the association between serum CTGF levels and SCD was not limited to higher CTGF levels, developing a comprehensive risk scoring system is imperative.

In the secondary analysis, an integrated multivariate risk score was developed using significant predictors from Cox regression (age, albumin, CTGF, NT-proBNP, hs-CRP, HD vintage, DM, smoking). Risk score stratification showed progressive increases in mortality across score categories. ROC analysis demonstrated strong predictive performance with AUCs of 0.907 for all-cause mortality, 0.883 for CV mortality, and 0.877 for SCD at 24 months (Figure 2). Compared with using CTGF or NT-proBNP alone, the integrated multivariate scoring model provided improved discrimination, particularly for SCD risk classification. Above results deserve further discussion.

## 3. Discussion

An optimal medical system of patient care requires early detection and continuous monitoring of high-risk individuals [7,19,20,21]. In MHD patients, the endless cycle of IDWG uremic toxin accumulation, and chronic inflammation predisposes them to great CV risks. Precise prognostic models integrating biological and clinical parameters are thus essential for improving therapeutic decision-making. A myriad of studies have integrated age, biomarkers, and clinical parameters into composite risk scores for high-risk CV populations [2,20,21]. Our findings emphasize that CTGF and NT-proBNP act as complementary biomarkers that reflect myocardial fibrosis, ventricular remodeling, and hemodynamic stress, providing a molecularly and clinically integrated framework for risk prediction.

CTGF has been identified as a potent prognostic biomarker in various populations, e.g., atherosclerosis, fibrosis, acute heart failure, cancer, neurological disorders and eye diseases [11,14,15]. Our findings align with these studies and demonstrate higher CTGF levels are associated with older age, longer HD duration, hypoalbuminemia, and increased risk of fatal events. Previous research using similar study methods supports our results [15], suggesting that patient heterogeneity and racial difference may not impact the model’s performance. Given that CTGF is a stress-induced protein, elevated circulating CTGF levels help physicians identify patients at high risk for various adverse clinical outcomes.

We introduce a composite scoring model that improves mortality risk prediction in MHD patients compared with single biomarkers. Because cardiorenal failure involves multiple comorbidities, single indicators cannot capture overall disease burden. This risk score can be integrated into routine monthly assessments to help clinicians identify high-risk patients early and implement timely interventions to improve outcomes [17,30]. The growing use of predictive modeling and risk score systems has the potential to strengthen patient safety. With recent advances in electronic medical records, machine learning, and statistical analytics, prediction models have become increasingly applicable in clinical care. Our death risk score system integrates clinical data with novel biomarkers to promote preventive healthcare. We anticipate its clinical implementation could assist clinicians and patients in adopting proactive, evidence-based management strategies [31,32,33,34].

From a molecular perspective, elevated CTGF levels may indicate activation of TGF-β/Smad and MAPK signaling cascades in response to mechanical stress, oxidative injury, and uremic toxin burden in MHD patients [35]. CTGF functions as a downstream effector that promotes fibroblast activation, α-SMA expression, and extracellular matrix (ECM) expansion, ultimately leading to ventricular stiffening [24,30]. Furthermore, CTGF-mediated activation of ILK/FAK–ERK1/2 signaling amplifies fibrotic remodeling and inflammation through ROS-dependent feedback mechanisms [36]. In parallel, upregulation of NT-proBNP represents a compensatory response to cardiac stretch and wall tension, mediated by the cGMP–PKG and calcineurin–NFAT pathways [26,28]. These molecular processes converge to form a mechano-fibrotic axis linking hemodynamic overload to adverse cardiac remodeling in MHD patients. The synergy between CTGF and NT-proBNP therefore offers a biologically plausible rationale for their combined use in mortality risk stratification. Our data demonstrated that CTGF and NT-proBNP are not only statistically significant but also biologically complementary predictors of mortality in MHD patients. Elevated CTGF levels reflect chronic activation of the fibrotic TGF-β/Smad axis and progressive extracellular matrix expansion, whereas NT-proBNP mirrors acute hemodynamic stress and cardiac overload. The integration of these biomarkers in a multivariate scoring system captures both structural and functional deterioration of the myocardium. This dual-axis model therefore provides a more complete picture of cardiac remodeling than either marker alone. In the future, similar models could be validated in larger sample size studies.

Comparatively, previous studies have predominantly relied on NT-proBNP as a solitary indicator of fluid overload and heart failure. However, such single-marker models are limited in their ability to distinguish between irreversible myocardial fibrosis and fatal arrythmia related SCD. In our study, CTGF significantly improved model performance, particularly in identifying patients prone to SCD despite optimized dialysis adequacy. The improved AUC for SCD prediction (composite risk score model 0.877 vs. NT-proBNP 0.863) underscores the prognostic value of integrating fibrotic signaling markers into conventional hemodynamic assessments. From a clinical standpoint, these findings suggest simultaneous quantification of CTGF and NT-proBNP could be incorporated into routine MHD monitoring protocols to facilitate individualized risk stratification. Monthly or quarterly assessment of both markers may help clinicians identify subclinical myocardial fibrosis or stress overload before overt cardiac decompensation occurs. Moreover, risk-based adjustment of dry weight, dialysis prescription, and cardioprotective therapy could further enhance patient survival. Future work should focus on external validation across larger multi-ethnic cohorts and longitudinal tracking of biomarker dynamics. Such studies may clarify whether decreasing CTGF or NT-proBNP levels over time reflect therapeutic efficacy, potentially guiding biomarker-based interventions and clinical decision algorithms in high-risk populations.

Beyond statistical validation, future work will focus on molecular and mechanistic verification using stored clinical serum specimens from MHD patients. By applying proteomic and transcriptomic analyses [37], it is worthwhile to identify downstream effectors and interacting partners of CTGF and NT-proBNP, including signaling molecules involved in TGF-β/Smad, MAPK, NF-κB, and oxidative stress pathways. Although these intracellular cascades are classically investigated in tissue samples, their systemic footprints can be captured in serum through circulating mediators and surrogate markers such as TGF-β1, CTGF, pro-inflammatory cytokines (IL-6, TNF-α), and oxidative stress indices (MDA, AOPP, SOD, GPx). Correlating these molecular signatures with patient outcomes will help establish a more direct causal link between circulating signals and underlying pathobiological processes. Such integrative serum-based validation may also uncover novel therapeutic targets and refine the prognostic model into a molecularly informed clinical tool for risk stratification in high-risk populations.

Several limitations of this study are noteworthy. One limitation of our study is the absence of detailed information on glycemic control among DM participants, which could confound the associations between CTGF, NT-proBNP, and CV mortality. Future studies incorporating markers such as HbA1c or time-in-range glucose monitoring are warranted. Next, the study’s sample size was relatively small, and many participants were Asian, which may limit the generalizability of our findings to other populations. Caution should be exercised when applying our model to different demographic groups. Secondly, the study relied on cross-sectional laboratory values, including CTGF levels, which may not capture intra-individual variability over time. This could affect the accuracy of our mortality predictions. Finally, due to limited sample size and statistical power, all cases of sudden death and CV events were grouped as cardiovascular mortality, preventing a more detailed analysis of specific events. We acknowledge that the pathophysiology underlying different CV outcomes may vary, and future studies with larger sample sizes could provide more comprehensive insights.

## 4. Materials and Methods

The primary goal of this study is to develop a cardiac multivariate risk score system for predicting all-cause, CV, and SCD in MHD patients. To achieve this, we first outline the dataset and participant cohort, including patient eligibility criteria and the bio-clinical parameters collected. We then describe the methods used to measure the plasma concentrations of CTGF and NT-proBNP, which form the basis of our risk score model. Following this, we explain how bio-clinical factors and covariates were integrated into the scoring system through Cox regression and ROC analysis. Finally, we detail the statistical analyses employed to validate our risk score system performance and draw a comparison of prediction values in different biomarker-based models.

### 4.1. Participants in the Cohort

The study design and methodology also have been mentioned in previous publications [20,38]. This research was conducted in accordance with the ethical standards of the declaration of Helsinki for medical research involving human subjects and approved by the institutional review board. Written informed consent was obtained from all participants. Eligibility criteria included patients undergoing MHD for a minimum of three months, and all participants were required to be at least 18 years of age and receive hemodialysis treatment three times a week. The total length of follow-up in the Cohort was 24 months. A total of 188 patients with end-stage kidney disease were initially included in the study. Twenty patients were excluded due to inadequate dialysis, terminal illness, active infections, advanced cancer, active hepatitis, severe protein-energy wasting, incomplete data, or unwillingness to participate (Supplementary Figure S1).

### 4.2. Assessment of Exposures

The plasma CTGF and NT-ProBNP levels were measured at baseline, along with bio-clinical parameters. Plasma concentrations of CTGF were measured using a commercial quantitative enzyme-linked immunosorbent assay kit, following the manufacturer’s instructions (Human CTGF/CCN2 DuoSet ELISA kit; DY9190-05 R&D, McKinley, MA, USA). NT-proBNP levels were measured with an electrochemiluminescence sandwich immunoassay. CTGF and NT-proBNP were combined as part of our myocardial remodeling-multivariate risk score model. In order to design a comprehensive scoring system, our mortality prediction model incorporated significant risk factors in the Cox regression model. Thus, we examined the associations between bio-clinical parameters, all-cause and CV mortality in the primary univariate Cox analyses. Significant predictors were used in the secondary ROC analyses. A published scale system was applied to determine cardiac biomarker-based multivariate death risk scores: The score (1 or 0) of the useful predictors was discriminated by their cut-off values in the ROC analysis [20]. We strengthen the power of validated predictor NT-proBNP: quartile 1 (the lowest concentration group) was scored as 0, quartile 2 was scored as 1, quartile 3 was scored as 2, and quartile 4 (the highest concentration group) was scored as 3. To exam the distribution, MHD patients were stratified into different risk categories based on their total scores. The predictive value of the total risk score system was evaluated for all-cause, CV mortality, and SCD using ROC analysis. Our stepwise approach aims to improve the risk score model’s effectiveness in predicting mortality outcomes, providing a robust tool for clinical decision-making.

### 4.3. Assessment of Covariates

Bio-demographic and clinical parameters were recorded for each MHD patient to assess their impact on mortality outcomes upon initial enrollment. The predictive values of parameters were specifically screened to form the cardiac multivariate risk score system: age, prior medical history, CTGF, NT-ProBNP, albumin, duration of hemodialysis (HD vintage), systolic and diastolic blood pressure, pre-dialysis blood urea nitrogen, glucose, creatinine, potassium, calcium, phosphorus, alkaline phosphatase, alanine aminotransferase, albumin, uric acid, total cholesterol, triglyceride, hematocrit, platelet, high-sensitivity C-reactive protein (hs-CRP), and intact parathyroid hormone. The HD vintage was defined as the duration of time between the first day of HD treatment and the first day that the patient entered the cohort. The blood pressure was recorded in the horizontal recumbent position before a midweek dialysis session. Pre-dialysis blood samples were obtained from the existing vascular access for further analyses. We adjusted plasma calcium according to the following equation: adjusted calcium = measured calcium + ((4-serum albumin in g/dL) × 0.8). All laboratory tests were performed by the standard procedures with certified methods [5,14,19,39]. Age was considered a critical factor, with patients over 65 years assigned an additional point in the multivariate risk score [2]. CTGF levels were measured and evaluated as a potential predictor of CV risk [14,15]. NT-proBNP levels were categorized into several tiers to assess the risk of heart failure and fluid overload, with higher levels indicating increased risk [18,40]. Albumin levels served as an indicator of nutritional status and inflammation, with low levels contributing to higher risk score values [38]. Prior medical history of CVD, hypertension, diabetes mellitus (DM) and smoking were each assessed as categorical variables, with the presence of either condition increasing the risk [5,39]. Prior CVD were defined as diseases attributable to myocardial ischemia and infarction (ICD-10-CM Diagnosis Code: I20–I25), heart failure (ICD-10-CM Diagnosis Code: I50.1–I50.9), symptomatic or life-threatening arrhythmia (ICD-10-CM Diagnosis Code: I49), cerebrovascular diseases (ICD-10-CM Diagnosis Code: I60–I69), pulmonary embolism (ICD-10-CM Diagnosis Code: I26), and peripheral artery diseases (ICD-10-CM Diagnosis Code: I73). These covariates were chosen to optimize the multivariate risk score system’s predictive accuracy for diverse fatal events in MHD patients. A more comprehensive integration into the risk score model optimizes a framework for assessing patient risk profiles and informing clinical decision-making.

### 4.4. Ascertainment of Outcomes

CV mortality among participants in the study was defined as death resulting from myocardial ischemia and infarction, heart failure, fatal arrhythmia, cardiac arrest due to other causes, cerebrovascular diseases, pulmonary embolism, peripheral artery diseases, and sudden otherwise unexplained death. SCD was specifically categorized as an unexpected death occurring within one hour of symptom onset or within 24 h of being last seen in a normal state, attributed to cardiac causes. Non-CV mortality included all other causes of death, such as infection, malignancies, gastrointestinal hemorrhage, accidents, and miscellaneous causes. All-cause mortality encompassed both CV and non-CV deaths. Patients were censored at their last follow-up appointment, if they switched to another dialysis unit, or if they received a kidney transplant.

### 4.5. Statistical Analysis

Continuous variables were expressed as mean ± standard deviation (SD), and categorical variables were presented as number (percentage). Univariate Cox regression analysis was performed to identify risk factors associated with all-cause, cardiovascular (CV) mortality, and SCD. Variables with statistically significant hazard ratios (HRs) in the univariate analysis were subsequently included in the multivariate Cox regression model and incorporated into the myocardial remodeling multivariate risk score system for mortality prediction.

Multivariable-adjusted hazard ratios (aHRs) for different mortality outcomes were calculated using the risk score system in the Cox regression model. Cumulative survival probabilities were estimated using Kaplan–Meier analysis and presented graphically, and the proportional hazards assumption was verified. The area under the receiver operating characteristic curve (AUC) was calculated to assess the discriminative ability of the risk score model for mortality outcomes. An AUC value of 0.5 indicates no predictive ability, whereas a value of 1.0 represents perfect prediction. A *p*-value < 0.05 was considered statistically significant. All analyses were conducted using PASW Statistics SPSS version 25.0 (IBM Corp., Armonk, NY, USA) and Python (version 3.11.11; Python Software Foundation, Wilmington, DE, USA) with the statsmodels package (version 0.14.4) for statistical modeling.

## 5. Conclusions

Risk stratification enables personalized care by guiding targeted interventions to improve outcomes. For MHD patients with high CV mortality, developing a robust death prediction model is in urgent need. Our study identifies CTGF and NT-proBNP as complementary biomarkers reflecting cardiac remodeling, fibrosis, hemodynamic instability and stress. The integrated multivariate risk score outperformed single predictors, especially for SCD, supporting its value as an early warning tool. Mechanistically, combining fibrosis-related (CTGF/TGF-β–Smad) and hemodynamic stress-related (NT-proBNP/cGMP–PKG) pathways provides a biologically grounded framework for mortality prediction. This model can guide cardiac screening, early intervention, and personalized therapy, with future studies aimed at multi-omics validation to enhance molecular precision in managing high-risk patients.

## Figures and Tables

**Table 1:** Comparisons of baseline bio-clinical characteristics according to circulating CTGF concentrations.

Variables	CTGF ≤ 30.2 ng/mL	CTGF > 30.2 ng/mL	*p*-Value
Diabetes Mellitus (n, %)	29 (35.8)	47 (54.0)	0.020
Cardiovascular Diseases (n, %)	28 (34.6)	49 (56.3)	0.005
Hypertension (n, %)	37 (45.7)	53 (60.9)	0.063
Smoking (n, %)	14 (17.3)	19 (21.8)	0.561
All-cause Mortality (n, %)	6 (7.4)	31 (35.6)	<0.001
Cardiovascular Death (n, %)	4 (4.9)	21 (24.1)	<0.001
Age (years ± std)	64.2 ± 6.9	73.6 ± 8.6	<0.001
Hemodialysis Vintage (months ± std)	66.6 ± 55.7	77.6 ± 42.4	0.150
Systolic Blood Pressure (mmHg ± std)	135.7 ± 21.1	138.8 ± 23.3	0.376
Diastolic Blood Pressure (mmHg ± std)	77.6 ± 10.0	78.4 ± 13.1	0.640
NT-ProBNP (pg/mL ± std)	475.7 ± 270.9	828.9 ± 370.9	<0.001
Albumin (g/dL ± std)	3.9 ± 0.4	3.8 ± 0.5	0.086
Alanine Aminotransferase (IU/L ± std)	12.6 ± 8.3	17.0 ± 14.3	0.016
Total Cholesterol (mg/dL ± std)	191.5 ± 50.2	190.1 ± 47.9	0.853
Triglycerides (mg/dL ± std)	243.4 ± 209.2	180.6 ± 156.4	0.028
Blood Urea Nitrogen (mg/dL ± std)	55.0 ± 17.0	63.0 ± 19.3	0.005
Creatinine (mg/dL ± std)	9.6 ± 2.1	10.1 ± 1.6	0.103
Blood Glucose (mg/dL ± std)	126.7 ± 43.5	141.2 ± 72.2	0.120
Uric Acid (mg/dL ± std)	7.3 ± 1.4	7.3 ± 1.1	0.813
Potassium (mmol/L ± std)	4.6 ± 0.9	4.4 ± 0.8	0.172
Calcium (mg/dL ± std)	9.2 ± 0.7	9.2 ± 0.7	0.811
Phosphate (mg/dL ± std)	4.4 ± 1.6	4.9 ± 1.5	0.062
Intact Parathyroid Hormone (pg/mL ± std)	229.3 ± 264.4	217.6 ± 220.3	0.755
Hematocrit (%) ± std	31.6 ± 3.9	31.9 ± 3.4	0.624
Platelet Count (k/μL ± std)	199.1 ± 69.6	197.6 ± 61.7	0.886
high-sensitivity C-Reactive Protein (mg/L ± std)	1.0 ± 0.4	1.7 ± 0.8	<0.001

Continuous data are presented as mean ± SD and categorical data as n (%). *p*-values were calculated using the independent *t*-test or Fisher’s exact test as appropriate. CTGF = Connective Tissue Growth Factor; NT-proBNP = N-terminal pro-brain natriuretic peptide.

**Table 2:** Potential prognostic parameters for various fatal outcomes in the univariate Cox regression analysis.

	All-Cause Mortality HR (95% CI)	*p*-Value	Cardiovascular Mortality HR (95% CI)	*p*-Value	Sudden Cardiac Death HR (95% CI)	*p*-Value
**Male**	0.836 (0.438–1.597)	*p* = 0.588	1.070 (0.488–2.345)	*p* = 0.866	0.676 (0.113–4.044)	*p* = 0.667
**Diabetes Mellitus**	2.653 (1.350–5.214)	*p* = 0.005	3.007 (1.296–6.973)	*p* = 0.010	5.177 (0.577–46.432)	*p* = 0.142
**Cardiovascular Diseases**	1.616 (0.846–3.086)	*p* = 0.146	2.900 (1.251–6.723)	*p* = 0.013	5.059 (0.565–45.301)	*p* = 0.147
**Hypertension**	1.082 (0.567–2.067)	*p* = 0.810	1.630 (0.720–3.690)	*p* = 0.241	1.322 (0.221–7.918)	*p* = 0.760
**Smoking**	2.143 (1.057–4.347)	*p* = 0.035	3.302 (1.479–7.370)	*p* = 0.004	6.656 (1.106–40.053)	*p* = 0.038
**CTGF (** **n** **g/mL)**	1.014 (1.005–1.022)	*p* = 0.002	1.015 (1.006–1.025)	*p* = 0.002	1.020 (1.001–1.040)	*p* = 0.042
**Age (years)**	1.065 (1.026–1.105)	*p* = 0.001	1.071 (1.023–1.121)	*p* = 0.003	1.273 (1.051–1.541)	*p* = 0.014
**Hemodialysis Vintage (months)**	1.007 (1.001–1.012)	*p* = 0.014	1.004 (0.997–1.011)	*p* = 0.232	0.998 (0.981–1.017)	*p* = 0.869
**Systolic Blood Pressure (mmHg)**	1.011 (0.997–1.026)	*p* = 0.135	1.023 (1.004–1.041)	*p* = 0.014	1.021 (0.980–1.063)	*p* = 0.322
**Diastolic Blood Pressure (mmHg)**	0.975 (0.948–1.004)	*p* = 0.086	0.979 (0.946–1.014)	*p* = 0.240	0.971 (0.900–1.048)	*p* = 0.452
**NT-ProBNP (pg/mL)**	1.003 (1.002–1.004)	*p* < 0.001	1.004 (1.003–1.005)	*p* < 0.001	1.003 (1.001–1.005)	*p* = 0.007
**Albumin (g/dL)**	0.191 (0.090–0.408)	*p* < 0.001	0.390 (0.153–0.997)	*p* = 0.049	0.586 (0.079–4.348)	*p* = 0.601
**Alanine Aminotransferase (IU/L)**	1.015 (0.993–1.038)	*p* = 0.181	1.021 (0.996–1.046)	*p* = 0.100	1.028 (0.980–1.078)	*p* = 0.258
**Total Cholesterol (mg/dL)**	0.996 (0.990–1.003)	*p* = 0.278	0.998 (0.990–1.006)	*p* = 0.662	0.995 (0.976–1.014)	*p* = 0.590
**Triglycerides (mg/dL)**	0.998 (0.995–1.000)	*p* = 0.108	0.997 (0.993–1.001)	*p* = 0.106	0.990 (0.975–1.005)	*p* = 0.208
**Blood Urea Nitrogen (mg/dL)**	1.009 (0.993–1.026)	*p* = 0.270	1.011 (0.991–1.031)	*p* = 0.300	0.982 (0.934–1.033)	*p* = 0.486
**Creatinine (mg/dL)**	1.027 (0.866–1.216)	*p* = 0.762	1.119 (0.909–1.379)	*p* = 0.289	0.678 (0.439–1.049)	*p* = 0.081
**Blood Glucose (mg/dL)**	1.002 (0.997–1.007)	*p* = 0.436	1.004 (0.998–1.009)	*p* = 0.168	0.999 (0.985–1.014)	*p* = 0.936
**Uric Acid (mg/dL)**	1.085 (0.848–1.388)	*p* = 0.516	1.091 (0.809–1.472)	*p* = 0.568	0.754 (0.355–1.605)	*p* = 0.464
**Potassium (mmol/L)**	0.740 (0.504–1.088)	*p* = 0.125	0.689 (0.430–1.105)	*p* = 0.122	0.728 (0.253–2.095)	*p* = 0.556
**Calcium (mg/dL)**	0.869 (0.548–1.377)	*p* = 0.549	0.672 (0.372–1.212)	*p* = 0.186	0.429 (0.098–1.867)	*p* = 0.259
**Phosphate (mg/dL)**	1.100 (0.910–1.330)	*p* = 0.325	1.050 (0.823–1.341)	*p* = 0.693	0.442 (0.193–1.013)	*p* = 0.054
**Intact Parathyroid Hormone (pg/mL)**	1.001 (1.000–1.002)	*p* = 0.101	1.000 (0.999–1.002)	*p* = 0.748	0.997 (0.990–1.004)	*p* = 0.409
**Hematocrit (%)**	1.032 (0.933–1.141)	*p* = 0.538	1.025 (0.906–1.159)	*p* = 0.700	1.254 (0.943–1.667)	*p* = 0.119
**Platelet Count (k/uL)**	1.004 (0.998–1.009)	*p* = 0.173	1.007 (1.001–1.013)	*p* = 0.029	1.002 (0.989–1.015)	*p* = 0.746
**high-sensitivity C-Reactive Protein (mg/L)**	3.888 (2.755–5.486)	*p* < 0.001	3.617 (2.366–5.530)	*p* < 0.001	3.405 (1.296–8.948)	*p* = 0.013

Boldface indicates significant predictors for fatal outcomes. CI = Confidence interval; CTGF = Connective Tissue Growth Factor; HR = Hazard ratio; NT-proBNP = N-terminal pro-brain natriuretic peptide.

**Table 3:** Multivariate Cox regression analysis of prognostic parameters for all-Cause and cardiovascular mortality.

	All-Cause Mortality aHR (95% CI and *p*-Value)		Cardiovascular Mortality aHR (95% CI and *p*-Value)	
Model 1				
CTGF (ng/mL)	1.012 (1.000–1.023)	*p* = 0.045	1.018 (1.004–1.032)	*p* = 0.010
NT-ProBNP (pg/mL)	1.003 (1.002–1.004)	*p* < 0.001	1.004 (1.003–1.005)	*p* < 0.001
Albumin (g/dL)	0.286 (0.133–0.616)	*p* = 0.001	0.608 (0.233–1.584)	*p* = 0.309
Model 2				
CTGF (ng/mL)	1.014 (1.003–1.026)	*p* = 0.014	1.019 (1.006–1.033)	*p* = 0.006
NT-ProBNP (pg/mL)	1.003 (1.002–1.004)	*p* < 0.001	1.004 (1.003–1.005)	*p* < 0.001
Diabetes Mellitus	1.751 (0.875–3.505)	*p* = 0.113	1.889 (0.796–4.484)	*p* = 0.149
Model 3				
CTGF (ng/mL)	1.014 (1.003–1.026)	*p* = 0.016	1.018 (1.005–1.032)	*p* = 0.008
NT-ProBNP (pg/mL)	1.003 (1.002–1.004)	*p* < 0.001	1.004 (1.003–1.005)	*p* < 0.001
Hemodialysis Vintage (months)	1.004 (0.997–1.011)	*p* = 0.236	1.000 (0.992–1.009)	*p* = 0.912

CTGF = Connective Tissue Growth Factor; NT-proBNP = N-terminal pro-brain natriuretic peptide. Adjusted hazard ratios (aHR) and 95% CIs for predictors of all-cause and cardiovascular mortality in hemodialysis patients.

## Data Availability

The numeric data used to support the findings of this study are available from the corresponding author, J.-F.C., upon reasonable request.

## Supplementary Materials

The following supporting information can be downloaded at: https://www.mdpi.com/article/10.3390/ijms262311350/s1.
