# Supplementary material for: CCN2/CTGF-Driven Myocardial Fibrosis and NT-proBNP Synergy as Predictors of Mortality in Maintenance Hemodialysis
Source: Int J Mol Sci. 2025 Nov 24;26(23):11350. doi: 10.3390/ijms262311350 (PMC12692257; doi:10.3390/ijms262311350)
Supplement: Supplementary file 1 [file ijms-26-11350-s001.zip › ijms-3976814-Table S1.pdf]

Supplementary Materials

Table S1. Comparison of Bio-Clinical Parameters and Fatal Events According to the Multivariate Risk Score System in the Entire Study Population.

	**0-2**	**3-4**	**5-6**	**7-8**	**9-10**
**Patients, *n* (%)**	**48 (28.6%)**	**41 (24.4%)**	**33 (19.6%)**	**36 (21.4%)**	**10 (6.0%)**
**All-cause death (n, %)**	0 (0.0%)	0 (0.0%)	8 (24.2%)	22 (61.1%)	7 (70.0%)
**CV death (n, %)**	0 (0.0%)	0 (0.0%)	4 (12.1%)	16 (44.4%)	5 (50.0%)
**SCD (n, %)**	0 (0.0%)	0 (0.0%)	1 (3.0%)	3 (8.3%)	1 (10.0%)
**Male (n, %)**	29 (60.4%)	17 (41.5%)	13 (39.4%)	17 (47.2%)	6 (60.0%)
**Age (years)**	63.4 ± 5.5	65.7 ± 8.6	72.3 ± 8.3	74.3 ± 8.1	80.6 ± 5.8
**Diabetes Mellitus (n, %)**	11 (22.9%)	17 (41.5%)	13 (39.4%)	26 (72.2%)	9 (90.0%)
**Cardiovascular Diseases (n, %)**	15 (31.2%)	17 (41.5%)	19 (57.6%)	18 (50.0%)	8 (80.0%)
**Hypertension (n, %)**	26 (54.2%)	21 (51.2%)	20 (60.6%)	17 (47.2%)	6 (60.0%)
**Hemodialysis Vintage (months)**	56.0 ± 42.7	66.2 ± 53.7	76.3 ± 53.0	95.9 ± 47.8	77.8 ± 15.8
**Systolic Blood Pressure (mmHg)**	135.2 ± 19.5	135.2 ± 20.7	134.3 ± 26.1	144.6 ± 22.9	139.5 ± 23.4
**Diastolic Blood Pressure (mmHg)**	79.6 ± 9.0	78.5 ± 10.5	76.6 ± 12.8	75.6 ± 14.1	82.2 ± 13.5
**NT-PROBNP (pg/mL)**	354.2 ± 79.9	459.2 ± 235.8	778.0 ± 332.3	1079.5 ± 258.2	1027.8 ± 166.8
**Albumin (g/dL)**	4.1 ± 0.4	3.9 ± 0.3	3.9 ± 0.4	3.7 ± 0.4	3.3 ± 0.6
**CTGF (ng/mL)**	16.1 ± 9.7	31.9 ± 21.3	52.9 ± 35.6	51.6 ± 25.8	70.1 ± 27.4
**Aspartate Aminotransferase (IU/L)**	14.1 ± 5.3	15.9 ± 5.9	18.5 ± 9.4	17.8 ± 7.6	12.9 ± 3.8
**Alanine Aminotransferase (IU/L)**	13.8 ± 11.6	14.9 ± 11.5	14.4 ± 8.6	16.5 ± 16.3	15.5 ± 6.2
**Total Cholesterol (mg/dL)**	197.7 ± 47.4	187.0 ± 52.7	172.6 ± 38.3	201.8 ± 50.1	193.1 ± 57.2
**Triglycerides (mg/dL)**	246.4 ± 229.6	206.7 ± 162.4	176.4 ± 155.9	180.8 ± 110.9	279.5 ± 307.0
**Blood Urea Nitrogen (mg/dL)**	55.2 ± 15.2	56.1 ± 18.7	59.1 ± 17.0	66.6 ± 20.5	63.2 ± 25.1
**Creatinine (mg/dL)**	9.62 ± 2.23	9.80 ± 1.94	10.03 ± 1.76	9.89 ± 1.25	10.14 ± 2.06
**Blood Glucose (mg/dL)**	121.8 ± 38.1	135.5 ± 63.6	119.9 ± 47.1	152.0 ± 78.3	172.0 ± 76.8
**Uric Acid (mg/dL)**	7.4 ± 1.4	7.1 ± 1.3	7.2 ± 1.2	7.3 ± 1.1	7.6 ± 1.0

**Potassium (mmol/L)**	4.6 ± 0.9	4.6 ± 0.9	4.5 ± 0.7	4.3 ± 0.8	4.0 ± 0.9
**Calcium (mg/dL)**	9.3 ± 0.7	9.2 ± 0.7	9.2 ± 0.8	9.1 ± 0.7	9.2 ± 0.6
**Phosphate (mg/dL)**	4.4 ± 1.5	4.2 ± 1.6	4.8 ± 1.2	5.3 ± 1.4	4.8 ± 2.3
**Intact Parathyroid Hormone (pg/mL)**	202.1 ± 244.5	156.2 ± 99.6	242.9 ± 238.8	292.5 ± 319.9	285.3 ± 284.3
**Hematocrit (%)**	31.8 ± 4.4	31.5 ± 3.0	32.1 ± 2.6	31.5 ± 4.3	31.6 ± 3.7
**Platelet Count (k/μL)**	193.9 ± 70.6	193.7 ± 61.7	194.1 ± 53.9	201.9 ± 72.7	239.4 ± 65.9
**Smoking (n, %)**	5 (10.4%)	10 (24.4%)	5 (15.2%)	6 (16.7%)	7 (70.0%)
**high-sensitivity C-Reactive Protein (mg/L)**	0.93 ± 0.33	1.16 ± 0.52	1.25 ± 0.68	1.83 ± 0.75	2.79 ± 0.72

Continuous variables are expressed as mean ± standard deviation (SD), while categorical variables are shown as n (%).
