# Supplementary figures and images for: CCN2/CTGF-Driven Myocardial Fibrosis and NT-proBNP Synergy as Predictors of Mortality in Maintenance Hemodialysis
Source: Int J Mol Sci. 2025 Nov 24;26(23):11350. doi: 10.3390/ijms262311350 (PMC12692257; doi:10.3390/ijms262311350)

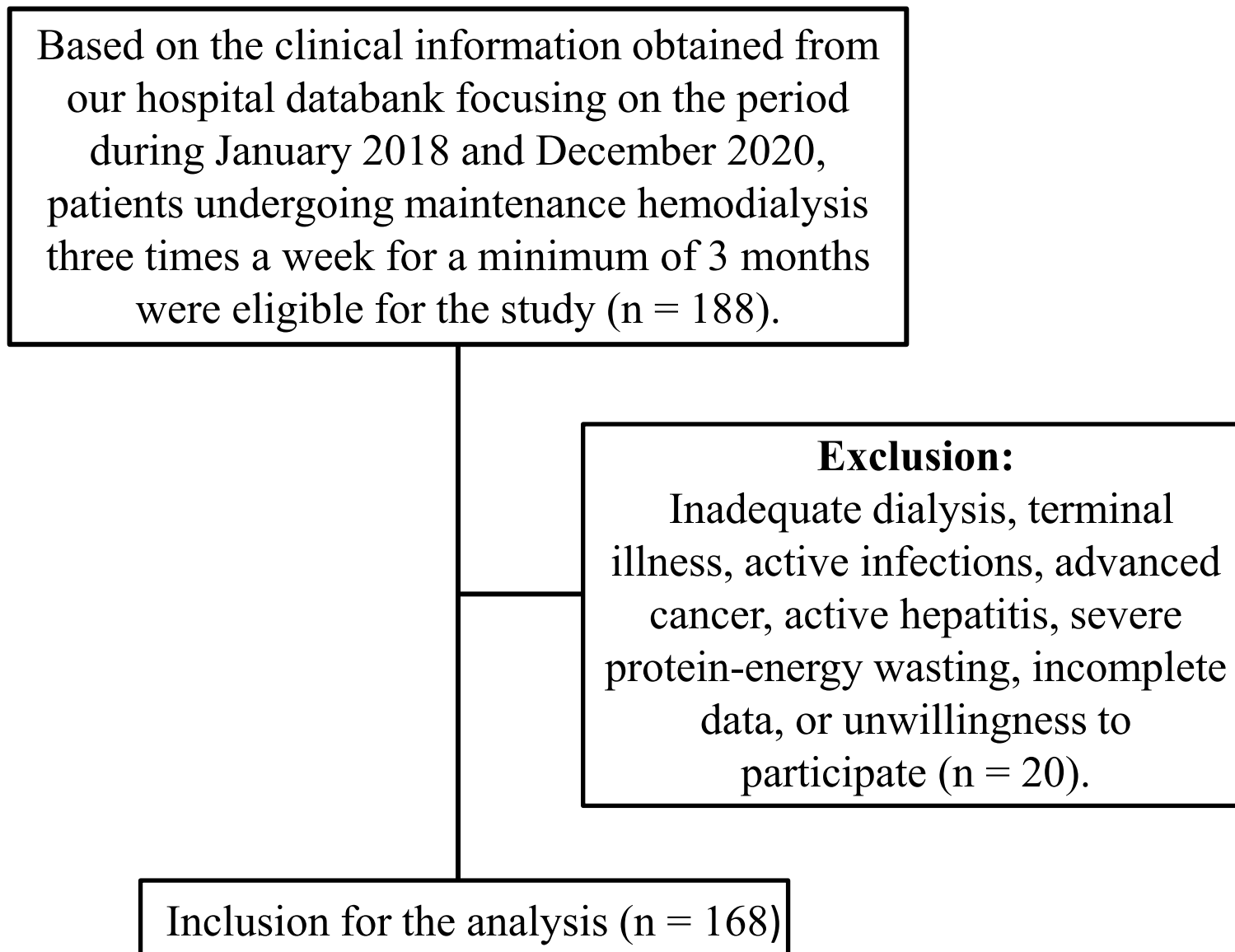

**Figure S1.** Patients with end-stage kidney disease.

Supplement: Supplementary file 1 [file ijms-26-11350-s001.zip › ijms-3976814-Figure S1.pdf]
